# Melatonin regulates mitochondrial dynamics and mitophagy: Cardiovascular protection

**DOI:** 10.1111/jcmm.70074

**Published:** 2024-09-27

**Authors:** Sohrab Rahmani, Ali Roohbakhsh, Vahid Pourbarkhordar, A. Wallace Hayes, Gholamreza Karimi

**Affiliations:** ^1^ Student Research Committee Mashhad University of Medical Sciences Mashhad Iran; ^2^ Department of Pharmacodynamics and Toxicology, School of Pharmacy Mashhad University of Medical Sciences Mashhad Iran; ^3^ Pharmaceutical Research Center Institute of Pharmaceutical Technology, Mashhad University of Medical Sciences Mashhad Iran; ^4^ Center for Environmental Occupational Risk Analysis and Management, College of Public Health University of South Florida Tampa Florida USA

**Keywords:** cardiovascular disease, dynamin‐related protein 1, heart, melatonin, mitochondrial fission, mitochondrial fusion, mitophagy

## Abstract

Despite extensive progress in the knowledge and understanding of cardiovascular diseases and significant advances in pharmacological treatments and procedural interventions, cardiovascular diseases (CVD) remain the leading cause of death globally. Mitochondrial dynamics refers to the repetitive cycle of fission and fusion of the mitochondrial network. Fission and fusion balance regulate mitochondrial shape and influence physiology, quality and homeostasis. Mitophagy is a process that eliminates aberrant mitochondria. Melatonin (Mel) is a pineal‐synthesized hormone with a range of pharmacological properties. Numerous nonclinical trials have demonstrated that Mel provides cardioprotection against ischemia/reperfusion, cardiomyopathies, atherosclerosis and cardiotoxicity. Recently, interest has grown in how mitochondrial dynamics contribute to melatonin cardioprotective effects. This review assesses the literature on the protective effects of Mel against CVD via the regulation of mitochondrial dynamics and mitophagy in both in‐vivo and in‐vitro studies. The signalling pathways underlying its cardioprotective effects were reviewed. Mel modulated mitochondrial dynamics and mitophagy proteins by upregulation of mitofusin, inhibition of DRP1 and regulation of mitophagy‐related proteins. The evidence supports a significant role of Mel in mitochondrial dynamics and mitophagy quality control in CVD.

## 1 INTRODUCTION

Cardiovascular disease (CVD) affects the heart, capillaries, arteries and veins.^1^, ^2^, ^3^ CVD and its complications, including ischemia–reperfusion (I/R), septic cardiomyopathies and atherosclerosis, are significant causes of death worldwide. Despite recent advances in treatment, CVD remains a leading public health challenge globally.^1^, ^2^, ^3^ The mitochondria, multifunctional double‐membrane organelles, play an essential role in cells by modulating calcium homeostasis, reactive oxygen species (ROS) production, apoptosis, cellular metabolism and adenosine triphosphate (ATP) production.^4^, ^5^, ^6^

The heart is one of the main energy‐demanding and mitochondrial‐enriched organs and, therefore, sensitive to mitochondrial impairment.^7^ Mitochondrial dysfunction plays a major role in CVD pathogenesis.^7^ The mitochondria are key regulators of heart function and cardiomyocyte viability.^8^ Mitochondrial homeostasis was first described in yeast in the 1990s.^9^ Structurally, the mitochondria are highly dynamic organelles that undergo fission and fusion.^5^ Fusion occurs when two mitochondria join, resulting in a single, larger mitochondrion, while fission is the opposite process.^5^ Excessive fission has been linked to mitochondrial impairments, mainly attributed to elevated stress levels leading to cell death.^5^ In conditions like I/R injury,^10^ chronic heart failure,^11^ cardiomyopathies^12^, ^13^ and doxorubicin cardiotoxicity^14^, ^15^ mitochondria in the heart undergo fission. Promoting mitochondrial fusion or inhibiting mitochondrial fission improves their function. Damaged mitochondria are removed by autophagy (mitophagy).^9^

These phenomena are necessary for proper mitochondria functioning, and their disruption can lead to various diseases.^3^, ^5^ Cardiovascular function and adaptation to pathological stressors require a healthy mitochondrial network.^5^, ^6^ Thus, mitochondrial dynamics and mitophagy are two phenomena that influence mitochondrial health, particularly in cardiomyocytes that are highly vulnerable to ATP depletion.^3^ Recent cardioprotective studies have focused on mitochondrial dynamics and mitophagy.

**FIGURE 1:**
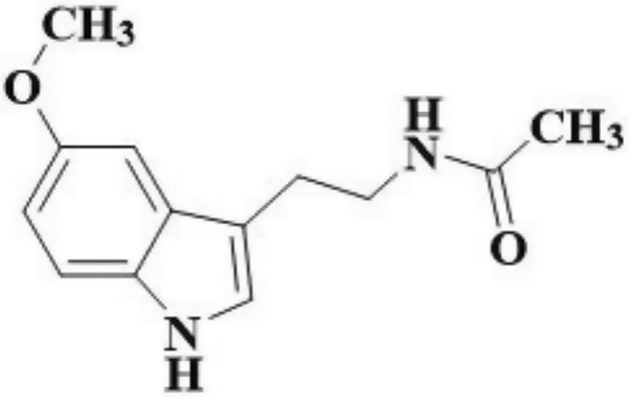
Chemical structure of melatonin.

Melatonin (Mel), N‐acetyl‐5‐methoxytryptamine (Figure 1), is a chronobiotic agent first identified in the cow pineal gland. Mel primarily regulates the sleep–wake cycle.^16^, ^17^, ^18^ Mel is also an effective anti‐oxidant, anti‐cancer and anti‐aging hormone.^17^, ^18^ The parent compound and several metabolites can also scavenge free radicals.^1^ Although the pineal gland is the principal site of Mel production and secretion,^17^ most organs and tissues can synthesize Mel, including the gastrointestinal tract, retina, ovary and heart.^1^

Plasma Mel levels are reduced in patients with coronary heart disease.^1^, ^10^ Exogenous Mel has been shown to improve systolic and diastolic blood pressure,^19^ maximum flow rate in the internal carotid artery^19^, ^20^ and decrease platelet aggregation. Accumulating evidence suggests a correlation between a reduction in the circulating level of Mel and its metabolite 6‐sulphatoxymelatonin with CVD such as congestive heart failure, nocturnal hypertension, coronary heart disease and myocardial infarction.^1^, ^21^

Mel functions via receptor‐ and non‐receptor‐mediated mechanisms.^1^ Receptor‐mediated effects of Mel are associated with its affinity for binding sites in the plasma membrane, cytoplasm and nucleolus. Mel targets some orphan nuclear receptors.^1^ Specific Mel receptors, MT1, MT2 and MT3, are distributed in cell membrane and nucleus thorough the body in places such as the brain, the retina of the eye and the cardiovascular system.^22^ MT1 and MT2 are membrane receptors coupled with G protein, while MT3 is a nuclear quinone reductase involved in oxidative stress reduction.^22^ Due to their high expression in ventricles, aorta, coronary arteries and endothelial cells, these receptors are potential targets for CVD treatment.^23^

Mel accumulates in mitochondria because of its amphiphilic nature and relatively small size.^24^ The concentration of Mel is higher in mitochondria than in blood.^25^, ^26^ Recent studies have suggested that the mitochondria can produce Mel.^26^, ^27^ The uptake of Mel by the mitochondria is mediated by the oligopeptide transporters GLUT/SLC2A10 and PEPT1/2 (SLC15A1/2),^25^, ^28^ suggesting that Mel specifically targets mitochondria.^25^ Although the molecular mechanisms involved in regulating mitochondrial dynamics and mitophagy are not fully understood, Mel has been reported to regulate mitochondrial dynamics in various cell lines and animal models of CVD.^29^, ^30^

Given the role of mitochondrial dynamics and mitophagy in CVD, we hypothesize that Mel may alleviate cardiovascular dysfunction by regulating mitochondrial dynamics and mitophagy. Therefore, this study evaluated the publicly available literature on the effects of Mel on mitochondrial dynamics and mitophagy in CVD in both in‐vivo and in‐vitro experimental models.

### 1.1 The role of mitochondrial fission in cardiovascular disease

In response to cardiovascular injuries, damaged mitochondria initially convert into small fragments through mitochondrial fission.^31^ Subsequently, excessive mitochondrial fission enhances ROS production. It promotes Bcl‐2‐associated X protein (Bax)/Bcl‐2 recruitment, oligomerization, pore formation at the outer mitochondrial membrane (OMM) and leakage of cytochrome c (Cyt c) into the cytoplasm. The mitochondrial fission‐dependent apoptosis is then triggered.^31^ Several proteins, including dynamin‐related protein (Drp1), fission factor 1 (Fis1), mitochondrial fission factor (MFF) and mitochondrial dynamics protein 49 and 51 (MiD49 and MiD51) regulate mitochondrial fission.^32^ In OMM, Fis1, MFF and MiD49/51 act as Drp1 receptors.^33^

Drp1, the master fission regulator, is a member of the GTPase superfamily with four main domains. These include C‐terminal, variable, helical middle and N‐terminal GTPase domains.^31^ Several studies have shown that Drp1 regulates mitochondrial fission. In addition, various post‐translational modifications control Drp1 activity during mitochondrial fission. These include SUMOylation, S‐nitrosylation, O‐GlcNAcylation, palmitoylation, ubiquitination and phosphorylation. Among them, phosphorylation plays a key role in regulating Drp1 activity. Phosphorylation occurs at different sites of Drp1 such as Ser‐40, Ser‐44, Ser‐579, Ser‐585, Ser‐616, Ser‐637 and Ser‐693. Ser‐616 and Ser‐637 have been extensively reported among these sites.^34^ Drp1 is activated by phosphorylation at Ser‐616 but inactivated by phosphorylation at Ser‐637.^35^ The balance of these two phosphorylation sites determines Drp1 activity.^35^ Activated Drp1 via phosphorylation migrates toward the surface of the mitochondria and at the predicted division sites along mitochondrial tubules.^35^ Several pathways have been reported to modify Drp1 activation, including c‐Jun N‐terminal kinases (JNK), extracellular signal‐regulated kinase (ERK) and protein kinase A (PKA).^5^ Excessive mitochondrial fission occurs within 60 min of myocardial reperfusion after transient ischemia, leading to mitochondrial dysfunction and decreased cardiac contractility.^36^

Inhibition of mitochondrial fission in CVD is a potential cardioprotective strategy. Researchers have investigated several Drp1 inhibitors, such as Mdivi‐1, Dynasore and P110. Mdivi‐1, a Drp1 inhibitor, has been extensively studied. While it is an efficient Drp1 inhibitor, it shows off‐target effects, such as complex I and complex II inhibition.^37^ Therefore, a primary challenge is finely modulating Drp1 activity while reducing possible side effects. Finding more selective Drp1 inhibitors is important to avoid potential off‐target side effects. Recent studies have investigated the role of specific inhibitors such as P110, which inhibits Drp1/Fis1 and P259, which inhibits Drp1/MFF. Targeting mitochondrial fission is a double‐edged problem because, physiologically, mitochondrial fission is required for mitochondrial function.

### 1.2 The role of mitochondrial fusion in cardiovascular disease

Mitochondrial fusion occurs when two mitochondria fuse to form one large mitochondrion. In the fusion, mtDNA is mixed, which may be beneficial. Proteins on the outer and inner membranes of the mitochondria mediate mitochondrial fusion. The key proteins that regulate the fusion of the mitochondrial outer membrane are mitofusins 1/2 (MFN1 and 2).^38^ Optic atrophy 1 (OPA1) is the key protein that regulates inner mitochondrial membrane fusion. Both belong to a family of dynamin‐related GTPases.^38^

The proteins involved in mitochondrial fusion have multiple cellular functions. For instance, OPA1, which exists in both long and short isoforms, is implicated in preserving crista structure and protecting cells from death stimuli.^38^ The optimum function of OPA1 depends on its protein expression level and a coordinated balance between its long and short isoforms.^38^ OPA1 knock‐out (KO) mice develop mitochondrial defects in the heart and late‐onset cardiomyopathy. Mutations in OPA1 in *Drosophila* have been associated with decreased heartbeat and cardiac arrhythmia.^39^ Charcot–Marie‐Tooth (CMT) and autosomal dominant optic atrophy (ADOA) are inherited neuropathies that lead to blindness. These diseases are associated with mutations in fusion proteins, MFN2 for CMT and OPA1 for ADOA.

### 1.3 The role of mitochondrial autophagy (mitophagy) in cardiovascular diseases

Excessive aberrant mitochondria can cause serious consequences, leading to cell death. The degradation of aberrant mitochondria through the autophagy pathway is termed mitophagy.^40^ Mitophagy is triggered by signals such as hypoxia, loss of mitochondrial membrane potential (MMP), opening of the mitochondrial permeability transition pore (mPTP) and excessive ROS production.^41^ Several pathways have been identified for mitophagy. Mitophagy is categorized into ubiquitin‐mediated and receptor‐mediated mitophagy. Ubiquitin‐mediated mitophagy includes PTEN‐induced kinase 1 (PINK1)/Parkin. The receptor‐mediated mitophagy pathways include Bcl‐2 interacting protein 3 (BNIP3)‐mediated mitophagy, FUN14 domain containing 1 (FUNDC1)−mediated mitophagy and lipid‐mediated mitophagy.^41^

PINK1 was identified in 2004 as a pathogenic gene of Parkinson's disease. PINK1 is a primary detector of mitochondrial damage.^42^, ^43^ It contains three domains: the N‐terminal domain, which is a mitochondrial target signal, the hydrophobic transmembrane domain and the C‐terminal domain.^42^, ^43^ PINK1 is regularly imported into normal healthy mitochondria through the N‐terminal domain and degraded by mitochondrial proteases and proteasomes.^42^, ^43^ In pathologic conditions, impairment in mitochondrial membrane potential prevents PINK1 from being imported from the cytosol into the mitochondria. This results in the PINK1 stabilization on the OMM in dimers and auto‐phosphorylation at Ser‐228 and Ser‐402.^44^ Activated PINK1 phosphorylates ubiquitin and activates Parkin, leading to mitophagy induction.^45^

In non‐conventional receptor‐mediated mitophagy, various OMM receptors have been identified, such as Bcl‐2 interacting protein 3 (BNIP3), FUNDC1 and BNIP3L/Nix, a pro‐apoptotic protein.^41^ BNIP3L is a mitophagy receptor that binds to Atg8 proteins.^46^ Phosphorylation and ubiquitination are the main post‐translational modifications of BNIP3L, which regulates BNIP3L‐mediated mitophagy. FUNDC1 is a newly discovered mitophagy receptor.^47^ FUNDC1 contains a LIR motif, three α‐helical stretches and a C‐terminal in the inner mitochondrial membrane. Dephosphorylation plays a key role in FUNDC1's binding affinity with LC3‐II. FUNDC1 and is deactivated when Src kinase and casein kinase 2 phosphorylate it at the tyrosine‐18 and Ser‐13 residues.^48^ Among these two sites, phosphorylation of tyrosine‐18 plays a central role, while phosphorylation of Ser‐13 provides a supplementary role in FUNDC1‐mediated mitophagy.^49^

Dysregulation of mitophagy has been implicated in CVD pathogenesis, such as atherosclerosis,^30^ I/R,^8^ cardiomyopathies^50^ and heart failure.^51^ The mitochondrial function in sustaining cardiomyocytes depends on mitophagy, which plays a dual role in the progression of heart diseases. Accordingly, it has been indicated that adaptive/mild mitophagy is associated with the alleviation of pathologies while maladaptive/excessive mitophagy could exacerbate pathological conditions.^3^ So, it seems that induction of mild mitophagy or decline in excessive mitophagy play a suitable strategy for CVDs. Therefore, cardiomyocyte mitophagy needs to be explored in more detail. It may provide theoretical and practical evidence to reduce heart injuries.

## 2 REGULATION OF MITOCHONDRIAL DYNAMICS AND MITOPHAGY BY MELATONIN IN HEART DISEASE

### 2.1 Myocardial ischemia/reperfusion

Ischemic heart disease is a major component of CVD and a leading cause of death.^21^ Although progress has been made in understanding the cause of ischemic heart disease, the underlying mechanism(s) that occurs during I/R injury have not been fully elucidated and current treatments are limited in effectiveness.^52^ Mitochondria play a central role in I/R pathogenesis.^8^ In this regard, the effects of Mel on I/R models in the setting of mitochondrial dynamics have been explored.^8^, ^20^, ^53^ The imbalance between mitochondrial fusion and fission contributes to cardiac dysfunction during I/R.^21^ During reperfusion, mitochondria undergo fission, while the fusion rate decreases.

Adenosine monophosphate‐activated protein kinase (AMPK), a serine/threonine protein kinase family member, regulates endogenous defensive molecules against various pathological processes in the heart.^54^ Evidence supports the promotive role of Mel on AMPK activation. Several dynamic processes of mitochondria, such as fusion, fission and mitophagy, are attributed to the AMPK signalling pathway.^5^, ^55^, ^56^ Moreover, the protective effects of Mel in in‐vivo and in‐vitro models have been attributed to the AMPK signalling pathway.^57^, ^58^ Zhou et al. found that Mel treatment ameliorated Drp1‐dependent mitochondrial fission through activation of AMPKα which subsequently influenced post‐transitional modification of Drp1 by increasing phosphorylation of Drp1 at Ser‐637 residue and decreasing phosphorylation of Drp1 at Ser‐616 residue^53^ (Table 1).

**TABLE 1:** Summary of the Mel‐induced protective effects on the cardiovascular system via modulation of mitochondrial fission/fusion in in‐vivo studies.

Cardiac disease Model	Animal	Toxic dose of agent, Duration, Route of exposure.	Dose of melatonin, Duration, Route of exposure	Finding(s)	Reference
Myocardial ischemia/reperfusion	Male Wistar rats	—	10 mg/kg/day, 16 weeks, oral	Decreased Mitophagy	24
	C57BL/6J mice	—	20 mg/kg, Single dose, i.p.	Increased Fusion Increased Mitophagy	8
	C57BL/6J mice	—	20 mg/kg, Single dose, i.p.	Increased Mitophagy	20
	Male Wistar rats	—	10 mg/kg, Single dose, i.v.	Decreased Fission Increased Fusion	60
	C57BL/6J mice	—	10,20 mg/kg, Single dose, i.p.	Increased Fusion	29
	C57BL/6J mice	—	20 mg/kg, Single dose, i.p.	Decreased Fission Decreased Mitophagy	53
	C57BL/6J mice	—	10 mg/kg/day, 60 days, oral	Increased Fission Increased Fusion	66
Myocardial infraction	C57BL/6J mice	—	10 mg/kg/day, 14 days, i.p.	Increased Fission	79
Doxorubicin cardiotoxicity	Male Wistar rats	3 mg/kg i.p. 6 days	10 mg/kg/day, 30 days, oral	Decreased Fission Increased Fusion	7
	Female Sprague Dawley rats	4 mg/kg, 3 days, i.p.	6 mg/kg, 14 days, oral	Decreased Fission Increased Fusion	99
Trastuzumab cardiotoxicity	Male Wistar rats	4 mg/kg/day, 7 days, i.p.	10 mg/ kg/day, 7 days, oral	Decreased Fission Increased Fusion	101
Diabetic cardiomyopathy	C57BL/6J mice	STZ 50 mg/kg/d, 5 days, i.p.	10 mg/kg, once daily, i.p	Decreased Fission Increased Fusion	67
	C57BL/6J mice	STZ 50 mg/kg/d, 5 days, i.p.	20 mg/kg, 28 days, oral	Increased Mitophagy	50
	Male Sprague Dawley rats	STZ 40 mg/kg, Single dose, i.p.	10 mg/kg/d,16 weeks, oral	Decreased Fission	71
Septic cardiomyopathy	C57BL/6J mice	LPS 15 mg/kg, Single dose, i.p.	20 mg/kg, Single dose, i.p.	Decreased Fission Increased Fusion Increased Mitophagy	73
	C57BL/6J mice	—	30 mg/kg, 3 doses, i.p.	Decreased Fission Increased Fusion Decreased Mitophagy	75
Post‐traumatic cardiac dysfunction	Male Sprague Dawley rats	—	30 mg/kg, Single dose, i.p.	Decreased Fission	77
Atherosclerosis	C57BL/6J mice	—	20 mg/kg/d, 28 days, i.p.	Increased Mitophagy	30
Cardiorenal syndrome type 3	C57BL/6J mice	—	20 mg/kg, Single dose, i.p.	Decreased Fission	102

The voltage‐dependent anion channel (VDAC1, mitochondrial porin) is localized on the OMM where its permeability is modulated by protein kinases such as glycerol kinase, creatine kinase and hexokinase.^57^ MMP reduction is the trigger for mitophagy activation. Mel treatment induced positive effects by mPTP opening suppression by enhancing the binding of hexokinase 2 to VDAC1. This decreases PINK1/Parkin‐mediated mitophagy.^53^ AMPK signalling pathway also contributes to signalling pathways of mitochondrial fusion^8^ (Table 1). Mel administration attenuated cardiac I/R damage and relieved mitochondrial stress by activating OPA1‐related mitochondrial fusion and mitophagy in an AMPK‐dependent manner.^8^ Mel treatment promoted mitochondrial fusion by enhancing OPA1 protein in cardiomyocytes in a hypoxia/reoxygenation model by increasing cell viability and reducing apoptosis and troponin c secretion. These effects were reversed by silencing the OPA1 gene^10^ (Table 2).

**TABLE 2:** Summary of the mel‐induced protective effects on the cardiovascular system via modulation of mitochondrial fission/fusion in in‐vitro studies.

Cardiac disease model	Experimental model	Toxic dose of agent Duration	Dose of melatonin, Duration	Finding(s)	Reference
In‐vitro studies					
Myocardial ischemia/reperfusion	H9C2 Cells	—	10 μM	Decreased Mitophagy	24
	Isolated perfused rat heart model	—	0.3,50 μM, 10 min	Decreased Fission Increased Mitophagy	36
	H9C2 Cells	—	1000 mM,15 min	Decreased Fission Increased Fusion	60
	H9C2 Cells	—	—	Increased Fusion	10
	Primary Cardiomyocytes	—	10,20 μM, 24 h	Increased Fusion	29
	Primary Cardiomyocytes	—	5 μM, 24 h	Increased Fusion	65
	H9C2 Cells	—	150 μM, 4 h	Increased Mitophagy	24
	H9C2 Cells	—	150 μM, 2 h	Decreased Fission Decreased Mitophagy	61
	CMEC Cells	—	5 μmol L, 12 h	Decreased Fission Decreased Mitophagy	53
	Isolated perfused rat heart model	—	50 μM, 15 min	Decreased Fission Increased Fusion	64
Diabetic cardiomyopathy	H9c2 Cells	—	100 μmol/L, 4 h	Decreased Fission Increased Fusion	67
	Primary neonatal cardiomyocyte culture	—	100 μmol/L, 4 h	Increased Mitophagy	50
Septic cardiomyopathy	Primary cardiomyocytes	LPS 5 μg/mL 48 h	10 μM	Decreased fission Increased Fusion Increased Mitophagy	73
Post‐traumatic cardiac dysfunction	H9C2 Cells	—	100 μmol/L	Decreased Fission	77
Atherosclerosis	RAW264.7 cells	ox‐LDL 50 mg/mL 24 h	10 μmol/L, 24 h	Increased Mitophagy	30
Chronic venous disease	HUVEC Cells	LPS 10 μg/m 12 h	20 μM, 12 h	Decreased fission	92
	HUVEC Cells	TNF‐α	Not mentioned	Decreased fission	103
Vascular Calcification	Isolated VSMC Cells	—	5 μmol/L, 14 days	Decreased fission	89
	Isolated VSMC Cells	—	5 μmol/L, 14 days	Increased Fusion Increased Mitophagy	90

Forkhead box O3a (FOXO3a), a member of the FOX family, is a transcription factor that directly regulates mitophagy by stimulating downstream targets like Parkin.^59^ By activating sirtuin 3 (SIRT3)/FOXO3a signalling via its receptor (MT2‐dependent), Mel curbed excessive mitophagy in H9c2 cells caused by anoxia/reoxygenation^24^ (Table 2). Singhanat et al. showed that Mel treatment at various time intervals (pretreatment, during ischemia and at the onset of reperfusion) exerted cardioprotective effects against cardiac I/R injury in rats. Mel treatment reduced excessive mitophagy, mitochondrial fission and fusion by activating the MT2 receptor^60^ (Table 2).

P62 is the link between LC3 and ubiquitin substrates. The increased expression levels of P62 were correlated with a decrease in mitophagy. Bai et al. demonstrated that Mel treatment reduced mitophagy by P62 upregulation in an anoxia/reoxygenation injury model. The results indicated that Mel post‐conditioning decreased mitophagy through the SIRT3/SOD2 signalling pathway^61^ (Table 2).

By regulating mitochondrial dynamics and homeostasis, the Yes‐associated protein (Yap)–Hippo pathway has been associated with cerebral and cardiac IR injury.^62^ Mel treatment can activate Yap protein, thereby stabilizing OPA1 and increasing mitochondrial fusion in an I/R model. However, the mechanism by which Yap controls OPA1 expression remains to be elucidated^29^ (Table 2).

MiRNAs are small noncoding regulatory RNAs that modify protein‐coding genes post‐transcriptionally.^63^ MicroRNAs are also implicated in some mitochondrial‐related processes, such as oxidative phosphorylation, lipid metabolism and mitochondrial dynamics.^64^ MicroRNA‐499 (miR‐499) is involved in the modulation of mitochondrial fission by modifying DRP1.^64^ The co‐treatment of nicotinamide mononucleotide and melatonin in an isolated I/R rat heart model increased microRNA‐499 expression levels, thereby influencing mitochondrial dynamics proteins by downregulation of DRP1 and upregulation of MFN2^64^ (Table 2).

Several non‐conventional mitophagy pathways have been identified. Compelling evidence suggests that a non‐conventional pathway distinct from the PINK1‐Parkin dependent mitophagy pathway plays a role during cardiomyocyte stress.^36^ Major components of this pathway are unc‐51 like kinase 1 (ULK1), Rab9, receptor‐interacting serine/threonine protein kinase 1 (Rip1) and Drp1. Mel treatment increased the phosphorylated form of ULK1 and upregulated Rab9 expression. Moreover, Mel reduced mitochondrial fission by increasing DRP‐1 phosphorylation at Ser637^36^ (Table 2).

Excessive platelet activation has been correlated with I/R injuries, and this activation is highly energy consuming and, therefore, relies on the function of mitochondria.^20^ A recent study explored the effects of Mel treatment on platelet activity in response to I/R damage.^20^ Loss of peroxisome proliferator‐activated receptor γ (PPARγ) is related to FUNDC1 dephosphorylation and activation of the mitophagy process.^20^ Mel treatment enhanced platelet activation by restoring PPARγ content in platelets, subsequently inhibiting FUNDC1‐required mitophagy^20^ (Table 1). In addition, the role of the peroxisome proliferator‐activated receptor‐gamma coactivator 1 alpha (PGC1α) pathway in I/R model has been investigated.^65^ Mel treatment upregulated MFN2 and OPA1, whereas deficiency in PGC1α abolished the upregulation of mitochondrial fusion proteins, indicating that mitochondrial biogenesis restored mitochondrial fusion in an I/R model^65^ (Table 2).

Aging is one of the primary risk factors for CVD, and mitochondrial dysfunction is a hallmark of aging.^66^ Mel treatment was studied in wild‐type C57BL/6J and NLRP3‐KO mice. Mitochondrial fusion proteins (MFN2 and OPA1) normally decline with aging, while Mel treatment significantly increased mitochondrial fusion^66^ (Table 1).

### 2.2 Cardiomyopathy

#### 2.2.1 Diabetic cardiomyopathy

Diabetes continues to increase worldwide.^67^ Cardiovascular complications are a primary cause of morbidity and mortality in diabetic patients.^67^ Diabetic cardiomyopathy (DCM) is a major chronic complication of diabetes.^67^ These complications are independent of hypertension and other heart diseases.^67^ Multiple factors are responsible for the pathogenesis of DCM. Factors including excessive mitochondrial ROS production and resultant mitochondrial dysfunction are pivotal in DCM.^67^ An imbalance in mitochondrial fission/fusion dynamics is an early component that increases mitochondrial ROS production and induces mitochondrial dysfunction.^67^ Aberrant mitochondria are responsible for excessive ROS generation and cell death‐inducing factors.^5^ Adequate clearance of aberrant mitochondria by mitophagy may prevent cardiomyocyte apoptosis.^68^ To explore the involvement of mitophagy in the pathogenesis of diabetic cardiomyopathy, Wang et al. showed that a serine/threonine‐protein kinase, mammalian sterile 20‐like kinase 1 (MST1), which is attributed to the pathogenesis of many cardiac diseases, was inhibited by Mel treatment. As a result, Mel activated Parkin and promoted mitophagy in a DCM animal model^50^ (Table 1). Peroxisome proliferator‐activated receptor gamma coactivator 1‐alpha (PGC‐1α) is a key protein involved in several mitochondrial functions.^69^, ^70^ Ding et al. reported that Mel treatment reduced Drp1‐mediated mitochondrial fission by activating the SIRT1/PGC‐1α signalling pathway.^67^ Reduction of mitochondrial fission by Mel was associated with a decrease in ROS generation and apoptosis, thereby improving mitochondrial cardiac function^67^ (Table 1). Long‐term Mel exposure decreased Drp1 phosphorylation at Ser‐616. Mel possibly enhances SIRT6 in a receptor‐dependent manner, and subsequently, SIRT6 activates AMPK/PGC‐1α/AKT signalling, inhibiting Drp1 phosphorylation^71^ (Table 1).

#### 2.2.2 Septic Cardiomyopathy

Septic shock is caused by a severe inflammatory response. Despite the therapeutic approaches used so far, sepsis is the leading cause of mortality and morbidity in hospitalized patients worldwide.^72^ Sepsis‐induced myocardial injury, a reversible form of cardiac depression, is characterized by cardiomyocyte death, oxidative stress, an excessive inflammatory response, microvascular vasospasms and ventricular systolic disorder. As a result of excess inflammation, cardiomyocytes are the primary targets of death in septic cardiomyopathy.^72^ At the molecular level, two signalling pathways are altered in response to septic shock: mitochondrial injury and endoplasmic reticulum (ER) stress.^72^ Mitochondrial dynamic imbalance plays a significant role in septic cardiomyopathy. Fission and fusion balance are potential therapeutic targets for preventing and treating septic cardiomyopathy.^72^ Receptor‐interacting protein kinase 3 (RIPK3) is a serine/threonine‐protein kinase closely associated with inflammation pathology.^73^ The therapeutic effects of Mel on the sepsis model showed that the imbalance of mitochondrial dynamics was reversed by Mel through the suppression of RIPK3 expression^73^ (Table 1). The nucleotide‐binding and leucine‐rich repeat pyrin domains have emerged as key mediators of pathological inflammation in many diseases and are potential drug targets.^74^ NLRP3 inflammasome seems to be inhibited by Mel, leading to decreased mitochondrial fission by Drp1 downregulation and increased mitochondrial fusion through MFN2 upregulation. Mel treatment modulated PINK1 and Parkin proteins^75^ (Table 1).

### 2.3 Post‐traumatic cardiac dysfunction

Mechanical trauma, such as that caused by motor vehicle collisions, natural disasters and wars, represents a major economic and medical burden.^76^ It is well recognized that mechanical trauma may cause direct heart damage, such as cardiac contusion.^76^ However, several studies have indicated that mechanical trauma often results in secondary cardiac dysfunction in the later period of trauma, even without direct cardiac injury during the early period.^76^ It is difficult to diagnose secondary cardiac dysfunction caused by mechanical trauma accurately because its clinical features are often obscure.^76^ Interestingly, after mechanical trauma induced‐cardiac injury, the plasma level of tumour necrosis factor‐α (TNF‐α) increased, which resulted in Drp1 translocation and initiation of mitochondrial fission. Anti‐TNF‐α reduced Drp1 translocation and prevented Drp1 phosphorylation at the Ser616 residue. Mel treatment also attenuated Drp1‐mediated mitochondrial fission through TNF‐α downregulation^77^ (Table 1).

### 2.4 Myocardial infarction

Myocardial infarction (MI) is a life‐threatening cardiovascular disorder that results mostly from the occlusion of a coronary artery, preventing blood flow to the myocardium. Dysregulation of mitochondrial dynamics and mitophagy are closely associated with MI damage.^78^, ^79^ The Notch signalling pathway is essential for cell communication. This pathway plays a key role in cardiomyocyte development and cardiac homeostasis^80^ and is regulated by ligand‐receptor interaction. The ligands are Delta‐like1,3,4 and Jagged1‐2 and the receptors are Notch1‐4.^80^ Mel exposure relieved MI damages by regulating the Notch1/MFN2 pathway^79^ (Table 1).

## 3 REGULATION OF MITOCHONDRIAL DYNAMICS AND MITOPHAGY BY MELATONIN IN VASCULAR DISEASES

### 3.1 Atherosclerosis

Atherosclerosis involves the accumulation of a sticky substance called plaque inside the arteries.^81^ Atherosclerosis may result in severe cardiovascular diseases such as ischemia, myocardial infarction and stroke.^81^ Proinflammatory components are released during plaque rupture and thrombosis. Inflammation is thought to be the primary cause of atherosclerosis. In atherosclerosis, mitochondrial dysfunction facilitates the inflammatory response and lesion development.^82^ Under pathological conditions, mitochondria in endothelial cells undergo fission.^83^

NLRP3 is a pattern recognition receptor in the innate immune system.^30^ The NLRP3 inflammasome has gained attention as a potential drug target for diseases underpinned by inflammation.^84^ Mel treatment induced Sirt3/FOXO3/Parkin‐mediated mitophagy and reduced ROS levels, leading to diminished NLRP3 inflammasome activation^30^ (Table 1).

### 3.2 Vascular calcification

Vascular calcification, defined by the deposition of calcium‐phosphate complexes in the vascular system, is mainly caused by medical conditions like hypertension, diabetes and chronic kidney disease. It is associated with significant cardiovascular impairments.^85^ Mitochondria play a key role in the pathogenesis of vascular calcification.^85^ High phosphate levels impair mitochondrial function in vascular smooth muscle cells. This dysfunction is demonstrated by low mitochondrial membrane potential and ATP level, excessive ROS production and induction of apoptosis. It has been shown that mitochondrial dynamics contribute to the underlying molecular mechanisms of vascular calcification.^85^, ^86^ Notably, imbalances in mitochondrial dynamics contributed to inorganic phosphate‐induced vascular smooth muscle cell (VSMCs) calcification by upregulating Drp1 expression and phosphorylation.^87^ This was further supported by a study showing that Drp1 was upregulated in osteogenic differentiation of primary human VSMCs.^88^ Chen et al. showed that Mel treatment reduced calcification by enhancing AMPK protein expression. This led to Drp1‐mediated mitochondrial fission inhibition and apoptosis suppression.^89^ Using compound C, it was possible to demonstrate the effects of Mel on the AMPK/Drp1 signalling pathway in the VSMC isolated from the aortas of Sprague Dawley rats' model.^89^ Chen et al. also demonstrated that Mel treatment increased OPA1 expression, enhanced mitochondrial fusion and mitophagy, and consequently attenuated vascular calcification, oxidative stress, inflammation and apoptosis.^90^ Knockout of OPA1 in VSMCs, however, abolished the effects of Mel on mitochondrial fusion/mitophagy.^90^ The results indicated that mitochondrial fusion/mitophagy activation was related to AMPK activation.^90^

### 3.3 Chronic Venous Disease

Chronic venous disease (CHVD) is a debilitating condition affecting millions worldwide.^91^ CHVD is caused by inflammation within the venous circulation and is subjected to increased hydrostatic pressure, resulting in increased ambulatory venous pressure.^91^ Along with being a multifactorial disease, the hallmark of CHVD pathophysiology is inflammation.^91^ Dysfunctional endothelium is the consequence of the inflammatory cascade, and it plays a pivotal role in CHVD development and progression.^91^ Several studies have demonstrated that mitochondrial function affects inflammation‐related cell damage. Endothelial dysfunction is caused by damaged mitochondrial mass caused by abnormal mitochondrial dynamics and/or decreased mitochondrial autophagy. Using a CHVD model, Caui and colleagues explored the possible role of mitochondrial fission in the inflammatory response.^92^ They showed that ROS overproduction contributed to DRP1 post‐transitional modification at Ser 616 residue.^92^ Since endothelial cells have lower mitochondrial content, it was suggested that the source of ROS overproduction in endothelial cells may be related to xanthine oxidase (XO).^92^ More importantly, they showed that the protective effects of Mel against LPS‐induced fission mediated apoptosis was mediated via increased AMPK activity which was accompanied by the upregulation of sarcoplasmic/ER Ca^2+^ ATPase 2 which is responsible for the reabsorption of Ca^2+^ into ER^92^ (Table 2).

## 4 REGULATION OF MITOCHONDRIAL DYNAMICS AND MITOPHAGY BY MELATONIN IN CARDIOVASCULAR TOXICITIES

### 4.1 Doxorubicin cardiotoxicity

Doxorubicin (Dox) is an anthracycline widely used alone or in combination for the treatment of various types of cancer, such as breast cancer, small‐cell lung cancer, acute lymphoblastic leukaemia and acute myeloid leukaemia.^63^, ^93^, ^94^, ^95^ Unfortunately, the clinical use of Dox is limited by its irreversible cardiotoxicity.^96^, ^97^ Dox‐induced cardiotoxicity is multifactorial and not completely understood. Ample evidence, however, suggests various mechanisms such as excessive ROS generation, apoptosis, disturbance of mitochondrial dynamics and dysregulation of mitophagy are associated with Dox‐induced cardiotoxicity.^5^, ^7^ Mel exerts its cardioprotective effects against Dox‐induced cardiotoxicity by reducing oxidative damage, inflammation and apoptosis.^54^, ^98^ Arinno et al. demonstrated that Dox‐treated rats exhibited mitochondrial dysfunction by increasing mitochondrial ROS generation, mitochondrial membrane depolarization (MMP) and impairments in mitochondrial dynamics proteins.^7^ Mel treatment upregulated proteins related to the fusion process (OPA1, MFN1 and MFN2) and downregulated mitochondrial fission protein (p‐Drp1^Ser616^/Drp1) in Dox‐induced cardiotoxicity in rats.^7^ Mel treatment not only reduced Dox cardiotoxicity but also enhanced the anticancer activity of Dox and suppressed the tumour's growth.^99^ Moreover, Mel treatment enhanced mitochondrial function and morphology. PGC‐1α is a regulator of energy metabolism and mitochondrial biogenesis.^99^ Dox treatment reduced PGC‐1α levels and subsequently abolished ATP levels.^99^ Since the correlation of mitochondrial dynamics with mitochondrial biogenesis was convincing, it was suggested that Mel enhanced mitochondrial biogenesis and dynamics via PGC‐1α regulation in rats^99^ (Table 1).

### 4.2 Trastuzumab cardiotoxicity

Trastuzumab, the humanized monoclonal antibody that specifically targets the human epidermal growth factor receptor 2 **(**HER2) receptor, is the first choice for HER2^+^ breast cancer. Unfortunately, cardiotoxicity has emerged as a significant side effect. The molecular mechanisms involved are poorly understood, but all converge on the mitochondria.^100^ A recent study showed that trastuzumab cardiotoxicity is associated with mitochondrial dynamics impairment and mitophagy. The results indicated that trastuzumab cardiotoxicity reduced key mitochondrial proteins such as MFN2 and OPA1. In contrast, the key mitochondrial fission protein p‐Drp1^Ser616^ was upregulated, indicating that trastuzumab cardiotoxicity leads to impairment of normal mitochondrial dynamics.^101^ Mel treatment reversed the imbalance of mitochondrial dynamics by increasing MFN2 and OPA1, while reducing the p‐Drp1^Ser616^/Drp1 ratio^101^ (Table 1).

## 5 CONCLUSION AND PROSPECTIVE

Mitochondria play a crucial role in all cells, including cardiomyocytes, which are particularly sensitive to mitochondrial dysfunction. Recent studies indicated that mitochondrial dynamics and mitophagy changes may impact cardiovascular biology. Dysregulation of mitochondrial dynamics and mitophagy have been linked to a variety of CVDs, such as I/R, cardiomyopathies, cardiotoxicities and vascular diseases. Mel has recently received attention for its potential to target mitochondria and as a potential CVD treatment. This potential has been supported by detecting Mel in isolated mitochondria and identifying specific transporters in mitochondria.

Mitochondria produce free radicals. Mel has both direct and indirect antioxidant effects because it is an electron‐rich molecule (Figure 1). Mel increased the activity of oxidative stress‐related genes, including SOD2, CAT, GPX, Nrf2 and HO‐1. Considering the effects of Mel against oxidative stress‐induced injuries in various in‐vivo and in‐vitro studies, the beneficial effects of Mel can be attributed to its role in mitochondrial function improvement by alleviating oxidative stress.

**FIGURE 2:**
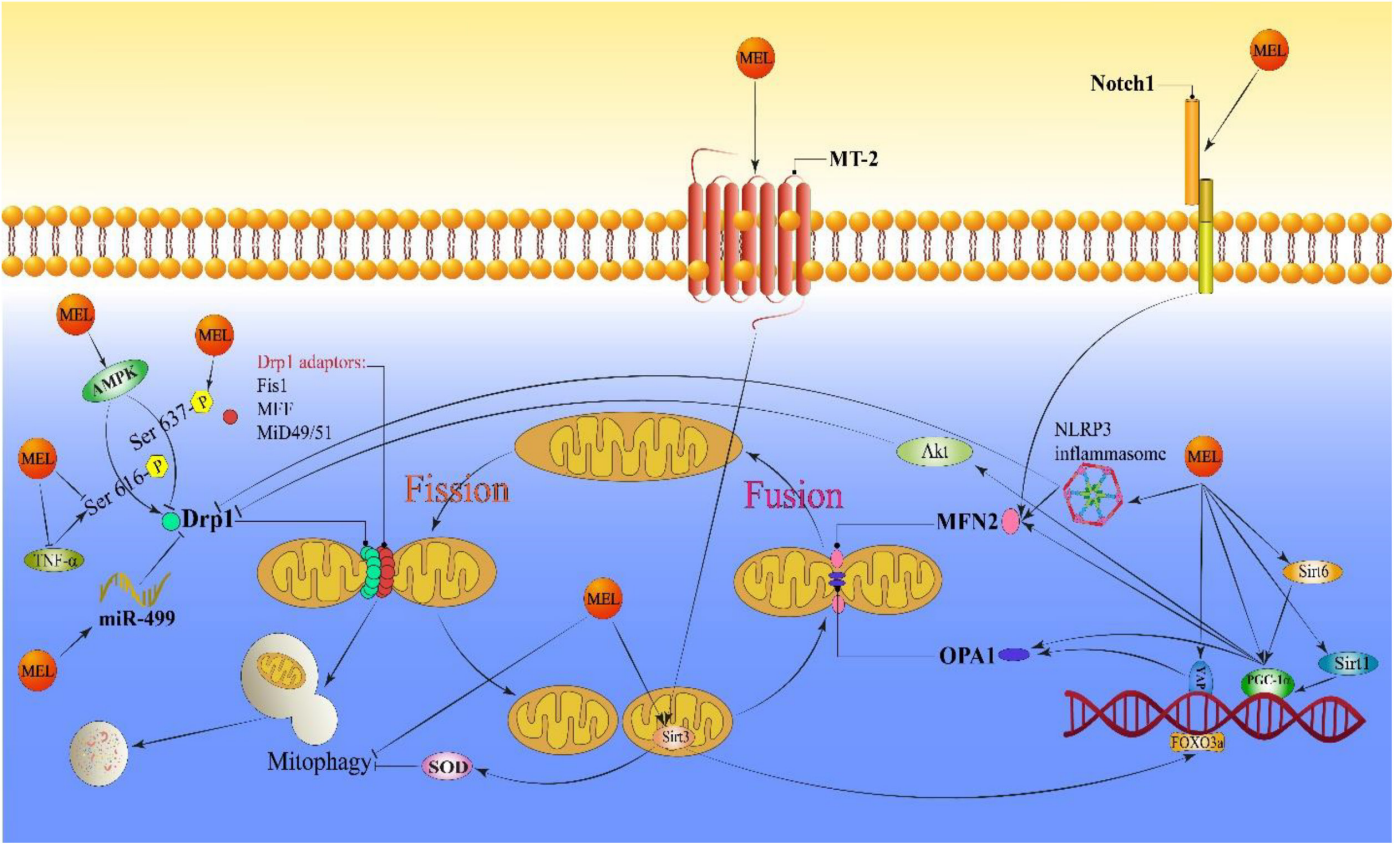
A schematic illustration of the Mel‐induced protective effects against CVDs damage through mitochondrial dynamics and mitophagy regulation. ↑ and → present the promote/activate, ⊥ and ↓ present the inhibitory/suppressive effects. AMPK, Adenosine monophosphate‐activated protein kinase, Drp1, dynamin‐related protein 1; MFN1, mitofusin‐1; MFN2, mitofusin‐2; NLRP3, nucleotide‐binding domain and leucine‐rich repeat pyrin domain containing 3; OPA1, optic atrophy 1; PGC1α, peroxisome proliferator‐activated receptor gamma coactivator 1‐alpha; Yap, yes‐associated protein.

Cardiomyocytes undergo mitochondrial fission in pathological conditions such as I/R, cardiomyopathies, cardiotoxicities and atherosclerosis. Mel mitigated mitochondrial fission via multiple pathways. Mel enhanced Drp1 activation by regulating AMPK, sirtuins and miR‐499 (Figure 2). Mel also exhibited cardioprotective effects by regulating mitochondrial fusion proteins and mitophagy. Mel treatment upregulated the mitochondrial fusion‐related proteins MFN1/2 and OPA1. Several reports have suggested that Mel's influence on the fusion rate was mediated by its effects on Notch‐1, YAP and AMPK (Figure 2).

Mel may be useful for treating some mitochondrial‐related heart diseases because it is well‐tolerated, less toxic and relatively inexpensive. Future studies should closely examine Mel's regulatory role on upstream pathways. A more detailed understanding of the molecular basis of the effects of Mel may contribute to developing specific therapeutic interventions for CVD patients.

## AUTHOR CONTRIBUTIONS

**Sohrab Rahmani:** Writing – original draft (equal). **Ali Roohbakhsh:** Writing – review and editing (equal). **Vahid Pourbarkhordar:** Writing – review and editing (equal). **A. Wallace Hayes:** Writing – review and editing (equal). **Gholamreza Karimi:** Conceptualization (equal).

## CONFLICT OF INTEREST STATEMENT

The authors declare no conflict of interest.

## Data Availability

Not applicable.
